# Post-Translational Modifications of Proteins Have Versatile Roles in Regulating Plant Immune Responses

**DOI:** 10.3390/ijms20112807

**Published:** 2019-06-08

**Authors:** Junjie Yin, Hong Yi, Xuewei Chen, Jing Wang

**Affiliations:** State Key Laboratory of Crop Gene Exploration and Utilization in Southwest China, Key Laboratory of Major Crop Diseases and Rice Research Institute, Sichuan Agricultural University at Wenjiang, Chengdu 611130, China; yinjunjie4154@163.com (J.Y.); hy190607@163.com (H.Y.); xwchen88@163.com (X.C.)

**Keywords:** plant immunity, post-translational modifications, signal transduction

## Abstract

To protect themselves from pathogens, plants have developed an effective innate immune system. Plants recognize pathogens and then rapidly alter signaling pathways within individual cells in order to achieve an appropriate immune response, including the generation of reactive oxygen species, callose deposition, and transcriptional reprogramming. Post-translational modifications (PTMs) are versatile regulatory changes critical for plant immune response processes. Significantly, PTMs are involved in the crosstalk that serves as a fine-tuning mechanism to adjust cellular responses to pathogen infection. Here, we provide an overview of PTMs that mediate defense signaling perception, signal transduction in host cells, and downstream signal activation.

## 1. Introduction

Plants are exposed to diverse types of pathogen during their lifetimes. To protect themselves from pathogen threats, plants have evolved effective immune systems that mediate microbe interactions. Several molecules are involved in plant immune systems. Pathogen-associated molecular patterns (PAMPs) are members of a large group of microbial molecules that include lipooligosaccharides of gram-negative bacteria, bacterial flagellin, elongation factor thermo unstable, glucans and glycoproteins from oomycetes, chitin from the fungal cell wall, and RaxX from *Xanthomonas oryzae* pv. *oryzae* (*Xoo*) [1,2]. Once PAMPs are recognized by plant cells, PAMP-triggered immunity (PTI), which is the first layer in plant innate immunity, is activated.

PAMPs are detected by pattern-recognition receptors (PRRs), which are normally surface-localized receptor kinases or receptor proteins [3,4]. However, only a handful receptor-like kinases (RLKs) and receptor-like proteins (RLPs) have been identified as being PRRs that perceive ligands in plants. A typical RLK should contain an extracellular domain (ECD) to bind the extracellular molecules, a transmembrane domain, and an intracellular kinase domain to activate signal transduction in cells [5]. In contrast, the intracellular domains of RLPs are very short and lack kinase activity [6,7]. The ECDs have various structures, including leucine-rich repeat (LRR) domains, lysin motifs, lectins, and epidermal growth factor-like domains. RLKs and RLPs can be divided into multiple subfamilies on the basis of ECDs [8]. As soon as the molecules are recognized by PRRs, the generation of reactive oxygen species (ROS), transcriptional reprogramming, and downstream gene activation occur subsequently [9,10,11].

Some evolved pathogens secrete effectors into the apoplast or directly into the cytoplasm of host cells, which can suppress PTI [12,13]. Conversely, plant cultivars have developed a family of polymorphic intracellular nucleotide-binding/leucine-rich-repeat (NLR) receptors that recognize particular effectors directly or indirectly, and induce the second major immune layer, called effector-triggered immunity (ETI), in order to stop pathogen growth [14,15]. The immune response elicited by ETI is very strong, typically accompanied with the programmed cell death of plant cells at the infection site, representing a hypersensitive response (HR) [16,17]. NLR receptors are structurally similar, containing an N-terminal coiled-coil or Toll-interleukin receptor domain, a central nucleotide-binding and a C-terminal LRR domain. The effectors recognized by NLR receptors are highly variable microbial molecules [18,19]. Therefore, the resistance mediated by ETI is predicted to be less durable and more specific than that of PTI.

Post-translational modifications (PTMs) of proteins are universal events in cell signaling networks, regulating protein stability, localization, activity levels, and interactions that help substrate proteins enter different pathways in order to activate signaling transduction [20]. At present, various types of PTMs have been identified. For, example, phosphorylation is a famous PTM that prefers to target the hydroxyl groups of hydroxyl amino acids, such as threonine (Thr), serine (Ser) and tyrosine (Tyr), but can also target unusual residues, such as hydroxy-proline [21]. It is a reversible process that regulates signal transduction in eukaryotic cells. Ubiquitination is a PTM that modulates protein stability or activity and plays important roles in various aspects of plant growth and development, including embryogenesis, floral development, plant senescence and disease resistance [22,23]. A group of small Ub-like modifiers (SUMOs) can target lysine residues of proteins to initiate SUMOylation [24]. SUMOylation significantly influences the transcriptional and epigenetic landscape in order to enhance stress tolerance and development [25]. In addition, cysteine proteases mediated by PTMs can cleave the peptide bonds of substrates, leading to the target’s rapid degradation [26]. Protein *N*-glycosylation may be a PTM that can remove glucose and some specific mannose resides in the endoplasmic reticulum [27]. Moreover, these PTMs are not independent and participate in crosstalk. In most cases, the initiation of a PTM is dependent on other PTMs interacting with the same protein. Here, we present recent progress in understanding PTMs in plant immune-related networks, which play important roles in mediating signal exchanges between microbes and hosts that eventually control associations.

## 2. Many Protein Effectors Function through PTMs of Host Proteins in Plant Cells

Effectors are typically secreted from pathogens and then translocate into host tissues and cells to mediate their interactions with hosts [28]. Recent advances suggest that effectors can be divided into non-protein and protein effectors. Non-protein effectors consist of chemical and sRNA effectors [29]. During the infection process, pathogens can secret a large array of chemical effectors, such as some toxins and hormones, to affect protein biosynthesis, fate and delivery [30,31,32,33,34]. Pathogen-derived sRNA effectors have important roles in host–microbe interactions. sRNA effectors can regulate the expression of multiple host genes involved in plant immunity through either transcriptional or posttranscriptional gene silencing [35,36,37]. However, the detailed molecular mechanisms of non-protein effectors remain elusive. Based on a working model, protein effectors are generally classified into two distinct groups: apoplastic protein effectors and cytoplasmic effectors. Apoplastic protein effectors work in the extracellular spaces of host tissues and are involved in immune evasion, host cell wall degradation and host proteolytic activity inhibition. Cytoplasmic effectors disrupt the activity of host proteins involved in various cellular signal regulatory processes [38]. In recent years, studies have been conducted to elucidate the functions of PTMs triggered by effectors.

The host proteins’ stability may be destroyed by effectors. The effector AvrPtoB is secreted from *Pseudomonas syringae* (*P. syringae*) pv. *tomato* DC3000 and contains a plant U-box (PUB) type E3 ubiquitin ligase domain in its carboxyl terminus. AvrPtoB interacts with diverse host proteins through its N-terminal region and ubiquitinates them, including some receptor kinases such as Pto, Fen, Flagelin Sensing 2 (FLS2), Chitin Elicitor Receptor Kinase 1 (CERK1), BRI1-associated receptor kinase1 (BAK1) and Bti9 [39,40,41,42,43], transcriptional coactivators, such as nonexpressor of pathogenesis-related (PR) genes 1 (NPR1) [44], and mitogen-activated protein kinase (MAPK) kinase (MAPKK), such as MKK2 [45]. AvrPtoB manipulates the host’s ubiquitin system and induces the degradation of its targets in order to suppress plant immunity, providing a typical model of effector-triggered host protein degradation. Several other effectors, like XopL, XopK and XopAE from different *Xanthomonas* strains, also have the E3 ubiquitin ligase activity that is required for ETI and full virulence [46,47]. XopD, another type III effector from *Xanthomonas euvesicatoria*, carries a C-terminal SUMO protease domain that can process the precursor of the small ubiquitin-related modifier SUMO or remove SUMO from its target proteins. Tomato ethylene response factor (ERF) SIERF4 is a substrate of XopD. XopD directly targets SIERF4 in the subnuclear foci to deSUMOylate, causing SIERF4 destabilization and ethylene production inhibition, which is required for ethylene-mediated immunity and symptom development [48]. In addition to SUMO protease activity, XopD has nonspecific DNA-binding activity within its helix-loop-helix domain and transcriptional repression ability within its two conserved ERF-associated amphiphilic motifs [49]. Some studies suggested that XopD can target some transcription factors, such as myeloblastosis 30 (MYB30), in order to inhibit transcriptional activity through its helix-loop-helix domain [50].

In addition to ubiquitinating host proteins for degradation, some effectors belong to a family of cysteine proteases that proteolytically cleave their targets, leading to the targets’ rapid degradation. AvRpt2 was one of the first cytoplasmic effectors that was demonstrated to act as a cysteine protease [51]. After being secreted from bacteria and delivered into host cells, AvRpt2 is auto-cleaved and enters an active state. The activated AvRpt2 interacts with, and directly cleaves RPM1-interacting protein 4 (RIN4), generating two cleavage products that are suppressors of PTI [52,53]. Several putative AvrRpt2 homologs in sequenced bacterial genomes may have similar functions on their targets [54]. Cytoplasmic receptor-like protein kinases (RLCKs) belong to a subgroup of RLKs that possess cytoplasmic kinase domains but lack extracellular ligand-binding domains. Studies of RLCK-associated components and targets indicate that RLCKs regulate various downstream signaling nodes to set a complex array of defense responses against microbial pathogens [55]. Some effectors suppress plant immunity by blocking the functions of RLCKs. The *P. syringae* effector AvrPphB was identified as a cysteine protease protein, which cleaves PBL1-like protein kinases belonging to the subfamily of RLCK VII and inhibits PTI signaling [56].

Phosphorylation is an important process in the activation of immune responses upon ligand binding. Effector XopAU is a catalytically active protein kinase conserved in various *Xanthomonas* species. XopAU can directly associate and phosphorylate MAPKK/MEKK2, contributing to the development of disease symptoms in pepper plants [57]. Unlike XopAU, effector proteins HopAI1 and HopAO have been identified as phosphatases that reduce the phosphorylation of their substrates. HopAI1 is another widely conserved *P. syringae* effector in both plant and animal bacterial pathogens that can inactivate MAPKs by removing the phosphate group through its unique phosphor-threonine lyase activity, resulting in enhanced disease susceptibility in plants [58]. Tyrosine phosphatase, HopAO1 can directly interact with FLS2 and EF-TU receptor (EFR). Consistent with these interactions, HopAO1 leads to a reduction in the phosphorylation of FLS2 and EFR upon ligand treatment [59].

During the process of plant-pathogen co-evolution, plants have evolved multiple receptors to recognize different effectors and activate plant defense responses. The effectors detected by ‘matching’ resistance proteins (named R protein) may also be avirulence (AVR) proteins [60]. AvrPto is a bacterial avirulence protein that confers disease resistance to tomato carrying the *Pto*R gene. Upon delivered into plant cells, AvrPto can localize at the plasma membrane and recruit two LRR-RLKs, FLS2 and EFR. Binding of AvrPto to FLS2 and EFR can inhibit their kinase activities, blocking downstream phosphorylation signal transduction [61]. *Xanthomonas campestris* pv. *campestris* type III effector AvrAC is an auridylyltransferase that adds uridine 5′-monophosphate to, and conceals, conserved phosphorylation sites in PBL2′s activation loop [62]. Uridylylated PBL2 (PBL2UMP) acts as a ligand to initiate an ETI response [63]. However, under natural selection, avirulence genes seem to change rapidly, allowing pathogens to diversify their effectors and escape recognition by the plant *R* genes. Therefore, studying the molecular mechanisms of Avr proteins and R proteins is a challenge.

## 3. PTMs of Receptor Complexes Initiate Plant Immune Signaling

Pathogen recognition by PRRs results in the activation of signaling pathways that induce defense reactions. Most known PRRs can recruit other LRR-RLKs to form a receptor complex. The binding of PAMPs to the extracellular domains of the PRRs leads to the dimerization or oligomerization of receptors and co-receptors, causing phosphorylation dynamics in host cells [20].

**Figure 1:**
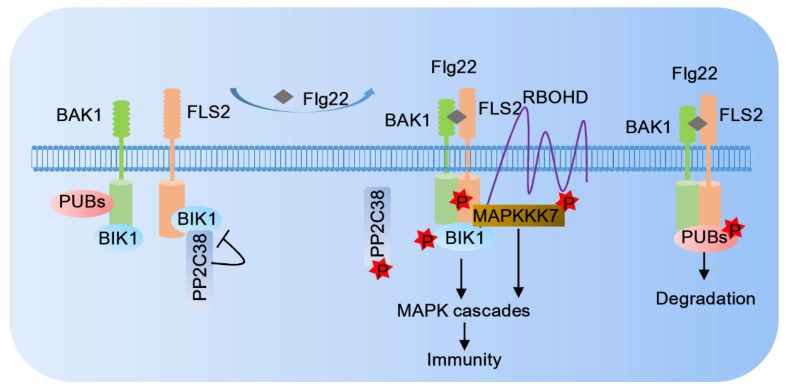
Ligand perception activates FLS2-mediated plant immunity. In the absence of flg22, BIK1 associates separately with FLS2 and BAK1 in an inactive state. Ligand perception induces rapid FLS2–BAK1 receptor complex formation and probably transphosphorylation, which further activates downstream intracellular immunity signaling.

One of the best characterized PRRs is the *Arabidopsis* LRR–RLK FLS2, which can recognize the bacterial PAMP flagellin (flg22 epitope) [9]. After flg22 perception, FLS2 forms a receptor complex with another LRR-RLK BAK1 and enters an activated state [64] (Figure 1). BAK1 is required for the immune responses mediated by multiple PRRs other than FLS2 [65]. The ligand-induced PRR-BAK1 complex formation can initiate phosphorylation/dephosphorylation between BAK1 and its interacting partners. Different phospho-patterns of BAK1 are associated with different RLKs, suggesting a phospho-code-based dichotomy of BAK1 functions in plant development and PRR-mediated immunity [66,67]. The activated PRR-BAK1 complex’s formation could initiate trans-phosphorylation between the receptor complex and receptor-like cytoplasmic kinases (RLCKs), such as Botrytis-induced kinase1 (BIK1). BIK1 can phosphorylate many PRRs and BAK1, and BIK1 is phosphorylated by BAK1 [46,68]. Phosphorylated BIK1 then directly interacts and phosphorylates NADPH oxidase, respiratory burst oxidase homolog protein D (RBOHD) and the phosphorylation of serine residues 39, 339, and 343 (S39, S339, S343) on RBOHD can modulate the ROS level [69,70]. BIK1 is not the only RLCKs involved in PRR-mediated immune signaling. Like BIK1, PTI-compromised receptor-like cytoplasmic kinase (PCRK) 1 and PCRK2 can interact with FLS2, and the flg22 treatment triggers PCRK2 phosphorylation to activate immune responses [71].

CERK1 is a Lys-RLK containing three extracellular LysM motifs and an intracellular Ser/Thr kinase domain, which is required for chitin- and peptidoglycan (PGN)-induced defense signaling [72]. In *Arabidopsis*, CERK1 works together with lysin motif receptor kinase 5 (LYK5), forming a receptor kinase complex to perceive chitin, and mutations in LYK5 result in a significant reduction in the chitin response [73]. Autophosphorylation of CERK1 triggered by chitin perception is essential for the activation of downstream responses. Like FLS2, some RLCKs, such as PBL27, are substrates of CERK1 that are required to transduce phosphorylation signals in plant immunity [74]. Unlike CERK1 in *Arabidopsis*, CERK1 in rice (*Oryza sativa*) cannot bind chitin molecules directly, despite the presence of extracellular LysM motif. Chitin elicitor-binding protein (CEBiP) is another LysM protein in the rice chitin receptor complex that can bind chitin with a high affinity [75]. Several RLCK VII members are immediate downstream signaling components of OsCERK1, including OsRLCK57, OsRLCK185, OSRLCK107, OsRLCK118 and OsRLCK176. OsRLCK118, OsRLCK176, and OsRLCK185 also emerged as substrates of other PRRs, including lysin motif-containing protein (LYP4), LYP6, and SPL11 cell-death suppressor2 [55,76], suggesting that the phosphorylation signaling triggered by PAMPs is transduced from PRRs located on the cell surface to cell cytoplasmic proteins dependent on these RLCKs.

Interactions between E3 ligase proteins and the kinase domains of RLKs appear to be a common feature of the regulation of various plant processes, including plant immunity. For example, two E3 ligases, PUB12 and PUB13, interact with the FLS2–BAK1 receptor complex, and the interaction between PUB12/13 and FLS2 is induced by flg22. PUB12 and PUB13 are substrates of BAK1, and the phosphorylation of PUB12/13 mediated by BAK1 is essential for the ubiquitination of FLS2 by PUB12/13. PUB12 and PUB13 ubiquitinate FLS2 for degradation, and flg22-triggered immunity responses are potentiated in the *pub12* and *pub13* mutants [77]. PUB12 and PUB13 ubiquitinate the brassinosteroid (BR) hormone receptor BRI1, which is important for the endocytosis and degradation of BRI1, a scenario that has been suggested for FLS2 [47]. In addition, PUB13 also ubiquitinates another chitin receptor LYK5. In the *pub13* mutant, the protein levels of LYK5 are constitutively enhanced [78]. XA21 is a rice RLK protein that confers resistance to *Xoo*. A RING-type E3 ubiquitin ligase, XA21-Bingding Protein3 (XB3), binds to the kinase domain of XA21 through an ankyrin repeat domain. XB3 is also a substrate for XA21 Ser and Thr kinase. Reducing the expression of *Xb3* increases resistance to *Xoo* and decreases the XA21 protein level, indicating that *Xb3* is necessary for the full accumulation of the XA21 protein and for Xa21-mediated resistance [79]. Like PRR activation, the ubiquitin-mediated regulation of PRRs is crucial for preventing the excessive or prolonged activation of immune responses.

Protein *N*-glycosylation is another major PTM in eukaryotic cells. Most PRRs are transmembrane glycoproteins that need to be transported by the secretory pathway to maturity and form their correct destination targets on the plasma membrane. Endoplasmic reticulum quality control (ERQC) is a pivotal process that assists these proteins to fold properly and avoid aggregation after they translocate into the ER. The *N*-glycosylation/ERQC pathway is necessary for the biogenesis and functions of several RLKs [80,81]. EFR is a typical example. EFR is specifically impaired in ERQC deficient mutants, including *stt3a*, *crt3*, *uggt*, *erd2b,* and *sdf2* [82,83,84]. Moreover, a single mutation at the conserved *N*-glycosylation site (N143Q) in the EFR ectodomain results in the loss of its ability to bind its ligand and to mediate elf18-elicited oxidative bursts [81]. However, the FLS2-mediated PTI response is not impaired in these ERQC mutants, suggesting that the folding of some PRRs is more complicated.

## 4. PTMs of RLCK–MAPK Are Required for Immune Signal Transduction

**Figure 2:**
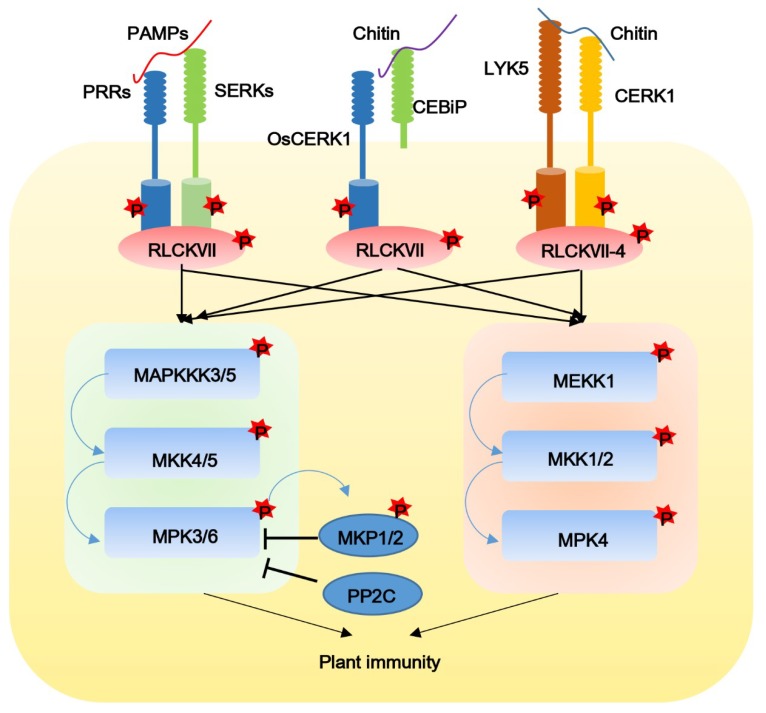
RLCKs and MAPK cascades function downstream of diverse PRRs to activate plant immune signals. Ligand perception induces rapid immune receptor complex formation and transphosphorylation. Several RLCK VII members rapidly become phosphorylated, which further activates evolutionarily conserved mitogen-activated protein kinase (MAPK) signaling modules, thereby activating pathway-specific transcription factors to activate plant immunity.

MAPK cascades represent signaling modules important for diverse intracellular immune responses in both PTI and ETI in plants [85]. A classic MAPK cascade has a set of three sequentially acting protein kinases, a MAPK, a MAPK kinase (MAPKK, MKK or MEK) and a MAPK kinase kinase (MAPKKK or MEKK), within which phosphorylation signals are transduced linearly from MAPKKK to MAPK [86]. Two MAPK cascades are activated by PRRs (Figure 2). One cascade is composed of the MAPKKK MEKK1, the two MAPKKs MKK1 and MKK2, and the MAPK MPK4. The other cascade consists of MAPKKK3 and MAPKKK5, the two MAPKKs MKK4 and MKK5, and the two MAPKs MPK3 and MPK6.

The first MAPK cascade, MEKK1-MKK1/2-MPK4 negatively regulates plant cell death and immunity. In *mekk1*, *mkk1/2* and *mpk4* mutants, programmed cell death and defense responses are constitutively activated [87,88,89,90]. MAPKKs, MKK1 and MKK2 can directly interact with both MEKK1 and MPK4 to form a special MAPK cascade. MPK4 is the substrate of MKK1/2 in vitro and in vivo, and MEKK1 and MKK1/2 are essential for the flg22-mediated activation of MAPK4 [88,90]. Interestingly, kinase inactivated MEKK1 (MEKK K361 M) rescues the autoimmune phenotype of *mekk1* and restores the flg22-induced activation of MPK4, suggesting that the MEKK1-activated MEKK1-MKK1/2-MPK4 pathway is independent of its kinase activity [90]. *Arabidopsis* SH4-Related3 functions as a transcriptional repressor and may be a substrate of MEKK1-MKK1/2-MPK4. It negatively regulates a large subset of flg22-induced genes through its ERF-associated amphiphilic repression motifs [91]. Another MAPK cascade, MAPKKK3/5-MKK4/5-MPK3/6 may be activated by the PRRs. It plays a far more important role in plant immunity by regulating defense hormones and phytoalexin synthesis and signaling, defense-related gene expression, ROS bursts and stomatal immunity [85]. Unlike the MEKK1-MKK1/2-MPK4 cascade, increasing evidence indicates that the MAPKKK3/5-MKK4/5-MPK3/6 cascade acts as positive regulator of defense responses. The MPK3/6 activation triggered by multiple patterns are greatly inhibited in *mapkkk3 mapkkk5* double mutants but slightly reduced in *mapkkk3* and *mapkkk5* single mutants [74,92,93]. Recently, other MAPK cascades have been found to play important roles in plant immunity. MKKK7 is a FLS2-interacting kinase and is rapidly phosphorylated on two different serine residues upon flg22 treatment. The *mpkkk7* mutant exhibits enhanced MPK6 activity, increased expression of defense genes, and increased ROS production [94]. The cascades mediated by MAPKKK7 remain elusive.

In addition to the PTMs of MAPK cascades mediated by effectors, several phosphatases that negatively regulate these MAPK cascades have been reported. Dual-specificity MAP kinase phosphatases (MKPs), such as MKP1 and MKP2, dephosphorylate both conserved Thr and Tyr residues of the MAPK activation loop, leading to the full inactivation of the MAPKs [95,96,97]. Furthermore, it is suggested that an efficient negative feedback loop exists between MKPs and MAPKs during PAMP responses and bacterial resistance [96]. Members of the protein phosphatase 2C (PP2C) family, AP2C1 and AP2C5, are classified as phosphor-Ser/Thr phosphatases that target a specific phosphor-Thr in the MAPK activation loop and negatively regulate PTI responses [98]. Loss of AP2C1 function results in an increased activation of MPK3, -4 and -6 in response to flg22 and elf18 treatments as well as enhanced callose deposition and bacterial resistance.

Upon PAMP recognition by RLK receptors, the receptor complex is activated, and immune signals are transduced into the nucleus by MAPK phosphorylation cascades, resulting in transcriptional reprograming for defense. As we just mentioned, RLCKs associate with, and rapidly become phosphorylated, by immune receptor complexes upon PAMP perception. RLCKs may fill the gap between the immune receptor kinase complex and the MAPK cascades [86,99]. In *Arabidopsis*, PBL27 associates with both CERK1 and MAPKKK5 on the plasma membrane. Chitin perception induces the CERK1-mediated phosphorylation of PBL27, which in turn phosphorylates MAPKKK5, leading to the activation of MPK3/6 through MKK4 and MKK5. The *mapkkk5* mutant displays compromised chitin-induced activation and disease resistance to *Alternaria brassicicola* [73,74,100,101]. Furthermore, Bi et al. found that MAKKK3/5-MAPK3/6 MAPK kinase cascades can be activated by multiple PRRs and confer resistance to bacterial and fungal pathogens in *Arabidopsis* [102]. Chitin induces the phosphorylation of MAPKKK5, and MEKK1 is largely disrupted in the *rlck vii-4* sextuple mutant. The RLCKVII-4 member, PBL9, can phosphorylate MAPKKK5 at Ser-599, which is required for MAPK activation and disease resistance [102]. The chitin-induced MAPK signaling pathway is conserved in rice and *Arabidopsis*. In rice, upon chitin perception, OsCERK1 phosphorylates OsRLCK185, an ortholog of PBL27 in *Arabidopsis*. Multiple MAPKKKs have been identified as substrates of OsRLCK185, including OsMAPKKK11, OsMAPKKK18 and OsMAPKKKε [103,104].

## 5. PTM Modulates TF Activities in Plant Immunity

Transcriptional reprogramming in response to a pathogen challenge is regulated by a broad variety of transcription factors (TFs) and cofactors in the nucleus. The study of TF regulatory mechanisms mediated by PTMs has increased our knowledge regarding the action of plant immunity. Among the 58 identified TF families in higher plants, six appear to be involved in defense signaling (Table 1) [105].

The ubiquitin proteasome system is responsible for controlling the stability and transcriptional activity levels of TFs involved in plant immunity. For example, MYB30 is a R2R3 MYB TF involved in pathogen-induced HR and cell death. Plants with a reduced *AtMYB30* expression level have a strongly compromised resistance to different bacterial strains [112]. A RING type E3 ubiquitin ligase, MYB30-interacting E3 ligase1 (MIEL1), interacts with and ubiquitinates MYB30, leading to MYB30 proteasomal degradation and the downregulation of its transcriptional activity. The *miel1* mutant plants show stronger HR cell death symptoms than Col-0 wild-type plants at 64 h post-infection with low dose of *P. syringae* pv. *tomato* DC3000 expressing the avirulence gene *AvrRpm1*. This is similar to *MYB30* overexpression plants [113,114]. In the AP2/ERF subgroup, another RING domain containing E3 ligase mediates AtERF53 for proteolytic degradation [115]. Similarly, the protein levels of many WRKY TFs are controlled by the ubiquitin proteasome system, including WRKY53 in *Arabidopsis*, and OsWRKY6, OsWRKY11, and OsWRKY45 in rice [116,117,118,119,120].

In response to pathogen attack, WRKYs are activated by MAPK cascades. WRKY33 is a substrate of MPK3/MPK6. A mutation of MPK3/MPK6 phosphorylation sites in WRKY33 compromises its ability to complement camalexin induction in the *wrky33* mutant [121]. In another MAPK cascade, MAPK4 substrate 1 (MKS1) acts downstream of MPK4 in a salicylic acid (SA)-dependent pathway. MKS1 interacts with WRKY 25 and WRKY33, and it regulates gene expression by releasing these TFs into the nucleus upon activation [122,123]. In tobacco, WRKY1 is a substrate of SA-induced protein kinase (SIPK). SIPK works as a MAPK to phosphorylate WRKY1, which results in enhanced the DNA-binding activity of WRKY1 to its cognate binding site, a W box sequence from the tobacco chitinase gene *CHN50* [124]. Phosphorylation may also be important for balancing rice yield and resistance. The *ideal plant architecture 1* (*IPA1*) gene encodes a kind of plant specific SQUAMOSA promoter-binding protein-like (SPL) TF, also known as *OsSPL14*, which activates yield-related genes [125]. Upon pathogen attack, IPA1 becomes phosphorylated at S163, which alters its DNA-binding specificity. IPA1(S163D) preferentially binds the WRKY45 promoter and activates WRKY45 expression, leading to enhanced resistance in *Magnaporthe oryzae*. However, IPA1 returns to the nonphosphorylated state within 48 h post-infection in order to active genes related to growth and high yield. Furthermore, the protein kinase regulating this process remains to be determined.

In addition to TFs, transcription cofactors play important roles in plant immunity. For example, NPR1 is hypothesized to be a transcription cofactor, contributing to the establishment of systemic acquired resistance (SAR), a mechanism of induced defense that is activated throughout a plant after exposure to various elicitors. NPR1 may also repress ETI by promoting programmed cell death (PCD) [126]. NPR1 is present as a homo-oligomer in a resting state that is dependent on its intermolecular disulfide bonds between cysteine residues. As the SA concentration increases in plant cells, NPR1 dissociates into mono molecules and is released into nuclei. NPR1 monomers in the nuclei prefer to interact with TFs and confer immunity through transcriptional cascades, resulting in the activation of *PR* genes and SA tolerance [127,128,129]. However, the expression of the *PR1* gene can be further upregulated in response to SAR induction in NPR1 cysteine mutants, such as *npr1C82A-GFP* and *npr1C216A-GFP*, suggesting that PTMs are involved in regulating the activity of NPR1 [129]. The proteasome-mediated degradation of NPR1 is required for its role in modulating the transcription of its targets. NPR1, containing a conserved broad-complex, tramtrack, and bric-à-brac/poxvirus, zinc finger (BTB/POZ) domain, is a substrate of CUL3-based ubiquitin ligase. In the absence of pathogen infection, the NPR4–CUL3-mediated degradation of NPR1 monomers is necessary to prevent the excessive activation of SAR and maintain plant development [130,131]. Recently, studies indicated that immune-induced transcriptome reprograming mediated by NPR1 requires the sequential actions of multiple E3 and E4 ligases [131]. In addition to ubiquitination, SUMOylation mediated by SUMO3 is also a regulatory step involved in the proteasome-mediated degradation of NPR1 [132]. Moreover, SUMOylation and phosphorylation work together to provide another regulatory layer that fine-tunes NPR1′s transcriptional activity. There are four putative phosphorylation serine residues on NPR1, Ser11, Ser15, Ser55 and Ser59. Under normal conditions, Ser55 and Ser59 of NPR1 are phosphorylated, which completely destroys the interaction between NPR1 and SUMO3. Non-SUMOylated NPR1 prefers to target WRKY70 to repress the expression of the *PR1* gene [132,133,134]. Upon pathogen challenge, Ser55 and Ser59 of NPR1 are rapidly dephosphorylated, allowing NPR1 to become SUMOylated. SUMOylated NPR1 can interact with TGA to promote the transcriptional activity of the *PR1* gene [132]. Furthermore, SUMOylation is required for SA-induced phosphorylation of Ser11 and Ser15 in NPR1, which accelerates the degradation of NPR1 facilitated by the NPR3–CUL3 E3 complex [132]. Interplay between phosphorylation and SUMOylation represents a perfect working model for NPR1′s regulation of plant immunity.

## 6. Conclusions

**Figure 3:**
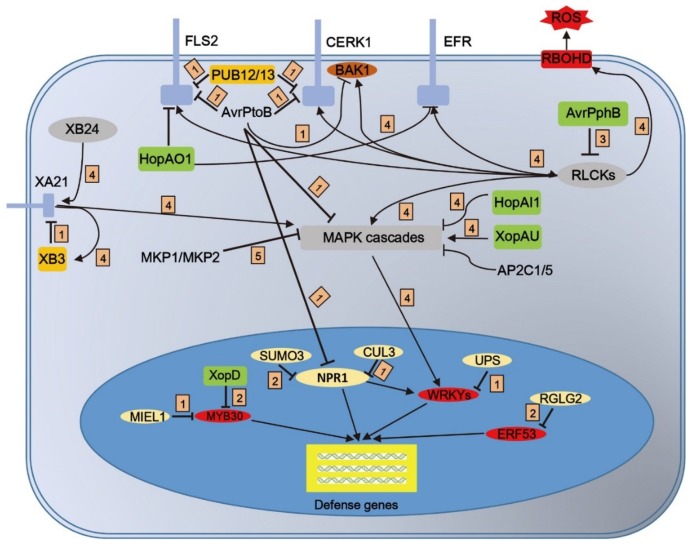
Schematic main PTMs in innate immunity of plants. Direct or indirect PTM activities plant immune response. PTM processes indicated are: 1, Ubiquitination/De-ubiquitination; 2, SUMOylation/De- SUMOylation; 3, cysteine protease; 4, Phosphorylation/De-phosphorylation.

PTMs are essential tools in plant defense signaling that allow plants to fight against pathogen invasions. Here, we have provided an overview of PTMs that modify components involved in plant defense signaling. PTMs establish communication between pathogens and plants to alter cell signaling at multiple nodes for the rapid reprogramming of the plant for defense responses (Figure 3). Within the developed proteomic technologies, PTMs that occur at a low stoichiometry are easier to detect. Their detection will accelerate our understanding of the regulatory mechanism of plant immunity mediated by PTMs.

Plant growth is usually inhibited by an active immune response, resulting in yield penalties for fighting pathogens. Plants with inactive immune responses may grow faster, but they will be more susceptible to various diseases [135]. Balancing development and resistance is a major challenge in crop breeding programs. PTMs, such as phosphorylation, may be involved in controlling the trade-off between plant development and resistance [66,67,136]. Gaining an in-depth understanding of how phosphorylation facilitates the balance between development and resistance, and whether other PTMs, such as ubiquitination and *N*-glycosylation, regulate these processes will provide new strategies for efficiently increasing plant resistance without fitness costs.

## Figures and Tables

**Table 1:** The major transcription factor (TF) families involved in plant defenses.

TF Families	Descriptions
AP2/ERF	The proteins in this family contain an AP2/ERF DNA-binding domain that consists of three β-sheet strands followed by an α-helix motif [106].
bHLH	This family is characterized by a basic-helix-loop-helix domain containing an N-terminal basic DNA-binding region and a C-terminal protein-interaction domain [107].
MYB	This family is characterized by the presence of four repeat sequences, each containing three α-helices [108]
NAC	NAC TFs each contain a conserved DNA-binding domain on their N-termini and an activation domain on their C-termini [109].
WRKY	The WRKY TFs contain WRKY domains having the typical WRKYGQK sequence followed by a zinc finger motif [110].
bZIP	The bZIP proteins contain a basic region for DNA binding and a leucine zipper region for protein dimerization [111]

## Abbreviations

AP2C1	*Arabidopsis* PP2C-type phosphatase
AVR	avirulence
BAK1	BRI1-associated receptor kinase1
BIK1	botrytis-induced kinase1
BR	brassinosteroid
BTB/POZ	broad-complex, tramtrack, and bric-à-brac/poxvirus, zinc finger
bZIP	basic leucine zipper
CEBiP	chitin elicitor-binding protein
CERK1	chitin elicitor receptor kinase 1
ECD	extracellular domain
EFR	EF-TU receptor
ETI	effector-triggered immunity
ERF	ET responsive TF
ER-QC	endoplasmic reticulum-quality control
FLS2	Flagellin Sensing2
HR	hypersensitive response
*IPA1*	*ideal plant architecture 1*
LRR	leucine-rich repeat
LYK5	lysine motif receptor kinase 5
LYP4	lysin motif-containing protein 4
MAMPs	microbe associated molecular patterns
MAPK	mitogen-activated protein kinase
MAPKK/MKK/MEK	MAPK kinase
MAPKKK/MEKK	MAPK kinase kinase
MIEL1	MYB30-interacting E3 ligase1
MKS1	MAPK4 substrate 1
MYB	myeloblastosis
NAC	no apical meristem, Arabidopsis transcription activation factor, cup-shaped cotyledon
NLRs	NOD-like receptors
NOD-like receptors	nucleotide-binding oligomerization domain-like receptors
*P. syringae*	*Pseudomonas syringae*
PAMPs	pathogen associated molecular patterns
PBL2	PBS1-like protein 2
PCD	programmed cell death
PCRK1	PTI compromised receptor-like cytoplasmic kinase1
PGN	eptidoglycan
PP2C	protein phosphatase 2C
PRRs	PAMPs are detected by pattern-recognition receptors
PTI	PAMP-/MAMP-triggered immunity
PTMs	post-translational modifications
PUB	plant U-box
RBOHD	respiratory burst oxidase homolog protein D
R protein	resistance protein
RLCKs	receptor-like cytoplasmic kinases
RIN4	RPM1-interacting protein 4
RLKs	receptor-like kinases
RLPs	receptor-like pro2teins
ROS	reactive oxygen species
RKS1	resistance-related kinase 1
SAR	systemic acquired resistance
Ser	serine
SERKs	somatic embryogenesis receptor kinases
SDS2	SPL11 cell-death suppressor 2
SIPK	SA-induced protein kinase
SPL	SQUAMOSA promoter-binding protein-like
SUMO	small Ub-like modifier
Thr	threonine
Tyr	tyrosine
XB3	XA21-Bingding Protein3
Xoo	*Xanthomonas oryzae* pv. *Oryzae*
ZAR1	HOPZ-activated resistance 1

## References

[1] Wei T., Chern M., Liu F., Ronald P.C. (2016). Suppression of bacterial infection in rice by treatment with a sulfated peptide. Mol. Plant Pathol..

[2] Zipfel C. (2014). Plant pattern-recognition receptors. Trends Immunol..

[3] Boutrot F., Zipfel C. (2017). Function, discovery, and exploitation of plant pattern recognition receptors for broad-spectrum disease resistance. Ann. Rev. Phytopathol..

[4] Choi H.W., Klessig D.F. (2016). Damps, mamps, and namps in plant innate immunity. BMC Plant Biol..

[5] Li L., Yu Y., Zhou Z., Zhou J.M. (2016). Plant pattern-recognition receptors controlling innate immunity. Sci. China Life Sci..

[6] Gust A.A., Felix G. (2014). Receptor like proteins associate with sobir1-type of adaptors to form bimolecular receptor kinases. Curr. Opin. Plant Biol..

[7] Liebrand T.W., van den Burg H.A., Joosten M.H. (2014). Two for all: Receptor-associated kinases sobir1 and bak1. Trends Plant Sci..

[8] Macho A.P., Zipfel C. (2014). Plant prrs and the activation of innate immune signaling. Mol. Cell.

[9] Gomez-Gomez L., Boller T. (2000). Fls2: An lrr receptor-like kinase involved in the perception of the bacterial elicitor flagellin in arabidopsis. Mol. Cell.

[10] Chinchilla D., Boller T., Robatzek S. (2007). Flagellin signalling in plant immunity. Adv. Exp. Med. Biol..

[11] Tsuda K., Sato M., Glazebrook J., Cohen J.D., Katagiri F. (2008). Interplay between mamp-triggered and sa-mediated defense responses. Plant J. Cell Mol. Biol..

[12] Fiorin G.L., Sanchez-Vallet A., Thomazella D.P.T., do Prado P.F.V., do Nascimento L.C., Figueira A.V.O., Thomma B., Pereira G.A.G., Teixeira P. (2018). Suppression of plant immunity by fungal chitinase-like effectors. Curr. Biol. CB.

[13] Houterman P.M., Cornelissen B.J., Rep M. (2008). Suppression of plant resistance gene-based immunity by a fungal effector. PLoS Pathog..

[14] Jones J.D., Dangl J.L. (2006). The plant immune system. Nature.

[15] Jacob F., Vernaldi S., Maekawa T. (2013). Evolution and conservation of plant nlr functions. Front. Immunol..

[16] Coll N.S., Epple P., Dangl J.L. (2011). Programmed cell death in the plant immune system. Cell Death Differ..

[17] Mur L.A., Kenton P., Lloyd A.J., Ougham H., Prats E. (2008). The hypersensitive response; the centenary is upon us but how much do we know?. J. Exp. Bot..

[18] Maekawa T., Kufer T.A., Schulze-Lefert P. (2011). Nlr functions in plant and animal immune systems: So far and yet so close. Nat. Immunol..

[19] Griebel T., Maekawa T., Parker J.E. (2014). Nod-like receptor cooperativity in effector-triggered immunity. Trends Immunol..

[20] Withers J., Dong X. (2017). Post-translational regulation of plant immunity. Curr. Opin. Plant Biol..

[21] Stulemeijer I.J., Joosten M.H. (2008). Post-translational modification of host proteins in pathogen-triggered defence signalling in plants. Mol. Plant Pathol..

[22] Shu K., Yang W. (2017). E3 ubiquitin ligases: Ubiquitous actors in plant development and abiotic stress responses. Plant Cell Physiol..

[23] Sharma B., Joshi D., Yadav P.K., Gupta A.K., Bhatt T.K. (2016). Role of ubiquitin-mediated degradation system in plant biology. Front. Plant Sci..

[24] Van den Burg H.A., Kini R.K., Schuurink R.C., Takken F.L. (2010). Arabidopsis small ubiquitin-like modifier paralogs have distinct functions in development and defense. Plant Cell.

[25] Augustine R.C., Vierstra R.D. (2018). Sumoylation: Re-wiring the plant nucleus during stress and development. Curr. Opin. Plant Biol..

[26] Grzonka Z., Kasprzykowski F., Wiczk W., Polaina J., MacCabe A.P. (2007). Cysteine proteases. Industrial Enzymes: Structure, Function and Applications.

[27] Roth J., Zuber C., Park S., Jang I., Lee Y., Kysela K.G., Le Fourn V., Santimaria R., Guhl B., Cho J.W. (2010). Protein n-glycosylation, protein folding, and protein quality control. Mol. Cells.

[28] Uhse S., Djamei A. (2018). Effectors of plant-colonizing fungi and beyond. PLoS Pathog..

[29] Collemare J., O’Connell R., Lebrun M.H. (2019). Nonproteinaceous effectors: The terra incognita of plant-fungal interactions. New Phytol..

[30] Lievens L., Pollier J., Goossens A., Beyaert R., Staal J. (2017). Abscisic acid as pathogen effector and immune regulator. Front. Plant Sci..

[31] Audenaert K., Vanheule A., Hofte M., Haesaert G. (2013). Deoxynivalenol: A major player in the multifaceted response of fusarium to its environment. Toxins.

[32] Wight W.D., Kim K.H., Lawrence C.B., Walton J.D. (2009). Biosynthesis and role in virulence of the histone deacetylase inhibitor depudecin from alternaria brassicicola. Mol. Plant Microbe Interact..

[33] Wicklow D.T., Jordan A.M., Gloer J.B. (2009). Antifungal metabolites (monorden, monocillins i, ii, iii) from colletotrichum graminicola, a systemic vascular pathogen of maize. Mycol. Res..

[34] Wang J., Huang Y., Fang M., Zhang Y., Zheng Z., Zhao Y., Su W. (2002). Brefeldin a, a cytotoxin produced by paecilomyces sp. And aspergillus clavatus isolated from taxus mairei and torreya grandis. FEMS Immunol. Med. Microbiol..

[35] Kusch S., Frantzeskakis L., Thieron H., Panstruga R. (2018). Small rnas from cereal powdery mildew pathogens may target host plant genes. Fungal Biol..

[36] Wang M., Weiberg A., Dellota E., Yamane D., Jin H. (2017). Botrytis small rna bc-sir37 suppresses plant defense genes by cross-kingdom rnai. RNA Biol..

[37] Weiberg A., Wang M., Lin F.M., Zhao H., Zhang Z., Kaloshian I., Huang H.D., Jin H. (2013). Fungal small rnas suppress plant immunity by hijacking host rna interference pathways. Science.

[38] Carella P., Evangelisti E., Schornack S. (2018). Sticking to it: Phytopathogen effector molecules may converge on evolutionarily conserved host targets in green plants. Curr. Opin. Plant Biol..

[39] Gohre V., Spallek T., Haweker H., Mersmann S., Mentzel T., Boller T., de Torres M., Mansfield J.W., Robatzek S. (2008). Plant pattern-recognition receptor fls2 is directed for degradation by the bacterial ubiquitin ligase avrptob. Curr. Biol. CB.

[40] Shan L., He P., Li J., Heese A., Peck S.C., Nurnberger T., Martin G.B., Sheen J. (2008). Bacterial effectors target the common signaling partner bak1 to disrupt multiple mamp receptor-signaling complexes and impede plant immunity. Cell Host Microbe.

[41] Gimenez-Ibanez S., Hann D.R., Ntoukakis V., Petutschnig E., Lipka V., Rathjen J.P. (2009). Avrptob targets the lysm receptor kinase cerk1 to promote bacterial virulence on plants. Curr. Biol. CB.

[42] Zeng L., Velasquez A.C., Munkvold K.R., Zhang J., Martin G.B. (2012). A tomato lysm receptor-like kinase promotes immunity and its kinase activity is inhibited by avrptob. Plant J. Cell Mol. Biol..

[43] Mathieu J., Schwizer S., Martin G.B. (2014). Pto kinase binds two domains of avrptob and its proximity to the effector e3 ligase determines if it evades degradation and activates plant immunity. PLoS Pathog..

[44] Chen H., Chen J., Li M., Chang M., Xu K., Shang Z., Zhao Y., Palmer I., Zhang Y., McGill J. (2017). A bacterial type iii effector targets the master regulator of salicylic acid signaling, npr1, to subvert plant immunity. Cell Host Microbe.

[45] Wei H.L., Chakravarthy S., Mathieu J., Helmann T.C., Stodghill P., Swingle B., Martin G.B., Collmer A. (2015). Pseudomonas syringae pv. Tomato dc3000 type iii secretion effector polymutants reveal an interplay between hopad1 and avrptob. Cell Host Microbe.

[46] Popov G., Majhi B.B., Sessa G. (2018). Effector gene xopae of xanthomonas euvesicatoria 85-10 is part of an operon and encodes an e3 ubiquitin ligase. J. Bacteriol..

[47] Qin J., Zhou X., Sun L., Wang K., Yang F., Liao H., Rong W., Yin J., Chen H., Chen X. (2018). The xanthomonas effector xopk harbours e3 ubiquitin-ligase activity that is required for virulence. New Phytol..

[48] Kim J.G., Stork W., Mudgett M.B. (2013). Xanthomonas type iii effector xopd desumoylates tomato transcription factor slerf4 to suppress ethylene responses and promote pathogen growth. Cell Host Microbe.

[49] Kim J.G., Taylor K.W., Hotson A., Keegan M., Schmelz E.A., Mudgett M.B. (2008). Xopd sumo protease affects host transcription, promotes pathogen growth, and delays symptom development in xanthomonas-infected tomato leaves. Plant Cell.

[50] Canonne J., Marino D., Jauneau A., Pouzet C., Briere C., Roby D., Rivas S. (2011). The xanthomonas type iii effector xopd targets the arabidopsis transcription factor myb30 to suppress plant defense. Plant Cell.

[51] Axtell M.J., Staskawicz B.J. (2003). Initiation of rps2-specified disease resistance in arabidopsis is coupled to the avrrpt2-directed elimination of rin4. Cell.

[52] Afzal A.J., da Cunha L., Mackey D. (2011). Separable fragments and membrane tethering of arabidopsis rin4 regulate its suppression of pamp-triggered immunity. Plant Cell.

[53] Coaker G., Falick A., Staskawicz B. (2005). Activation of a phytopathogenic bacterial effector protein by a eukaryotic cyclophilin. Science.

[54] Eschen-Lippold L., Jiang X., Elmore J.M., Mackey D., Shan L., Coaker G., Scheel D., Lee J. (2016). Bacterial avrrpt2-like cysteine proteases block activation of the arabidopsis mitogen-activated protein kinases, mpk4 and mpk11. Plant Physiol..

[55] Liang X., Zhou J.M. (2018). Receptor-like cytoplasmic kinases: Central players in plant receptor kinase-mediated signaling. Ann. Rev. Plant Biol..

[56] Zhang J., Li W., Xiang T., Liu Z., Laluk K., Ding X., Zou Y., Gao M., Zhang X., Chen S. (2010). Receptor-like cytoplasmic kinases integrate signaling from multiple plant immune receptors and are targeted by a pseudomonas syringae effector. Cell Host Microbe.

[57] Teper D., Girija A.M., Bosis E., Popov G., Savidor A., Sessa G. (2018). The xanthomonas euvesicatoria type iii effector xopau is an active protein kinase that manipulates plant map kinase signaling. PLoS Pathog..

[58] Zhang J., Shao F., Li Y., Cui H., Chen L., Li H., Zou Y., Long C., Lan L., Chai J. (2007). A pseudomonas syringae effector inactivates mapks to suppress pamp-induced immunity in plants. Cell Host Microbe.

[59] Macho A.P., Schwessinger B., Ntoukakis V., Brutus A., Segonzac C., Roy S., Kadota Y., Oh M.H., Sklenar J., Derbyshire P. (2014). A bacterial tyrosine phosphatase inhibits plant pattern recognition receptor activation. Science.

[60] Van de Wouw A.P., Cozijnsen A.J., Hane J.K., Brunner P.C., McDonald B.A., Oliver R.P., Howlett B.J. (2010). Evolution of linked avirulence effectors in leptosphaeria maculans is affected by genomic environment and exposure to resistance genes in host plants. PLoS Pathog..

[61] Zong N., Xiang T., Zou Y., Chai J., Zhou J.M. (2008). Blocking and triggering of plant immunity by pseudomonas syringae effector avrpto. Plant Signal. Behav..

[62] Feng F., Yang F., Rong W., Wu X., Zhang J., Chen S., He C., Zhou J.M. (2012). A xanthomonas uridine 5′-monophosphate transferase inhibits plant immune kinases. Nature.

[63] Wang G., Roux B., Feng F., Guy E., Li L., Li N., Zhang X., Lautier M., Jardinaud M.F., Chabannes M. (2015). The decoy substrate of a pathogen effector and a pseudokinase specify pathogen-induced modified-self recognition and immunity in plants. Cell Host Microbe.

[64] Chinchilla D., Zipfel C., Robatzek S., Kemmerling B., Nurnberger T., Jones J.D., Felix G., Boller T. (2007). A flagellin-induced complex of the receptor fls2 and bak1 initiates plant defence. Nature.

[65] Yasuda S., Okada K., Saijo Y. (2017). A look at plant immunity through the window of the multitasking coreceptor bak1. Curr. Opin. Plant Biol..

[66] Wang Y., Li Z., Liu D., Xu J., Wei X., Yan L., Yang C., Lou Z., Shui W. (2014). Assessment of bak1 activity in different plant receptor-like kinase complexes by quantitative profiling of phosphorylation patterns. J. Proteomics.

[67] Perraki A., DeFalco T.A., Derbyshire P., Avila J., Sere D., Sklenar J., Qi X., Stransfeld L., Schwessinger B., Kadota Y. (2018). Phosphocode-dependent functional dichotomy of a common co-receptor in plant signalling. Nature.

[68] Lu D., Wu S., Gao X., Zhang Y., Shan L., He P. (2010). A receptor-like cytoplasmic kinase, bik1, associates with a flagellin receptor complex to initiate plant innate immunity. Proc. Natl. Acad. Sci. USA.

[69] Kadota Y., Sklenar J., Derbyshire P., Stransfeld L., Asai S., Ntoukakis V., Jones J.D., Shirasu K., Menke F., Jones A. (2014). Direct regulation of the nadph oxidase rbohd by the prr-associated kinase bik1 during plant immunity. Mol. Cell.

[70] Li L., Li M., Yu L., Zhou Z., Liang X., Liu Z., Cai G., Gao L., Zhang X., Wang Y. (2014). The fls2-associated kinase bik1 directly phosphorylates the nadph oxidase rbohd to control plant immunity. Cell Host Microbe.

[71] Kong Q., Sun T., Qu N., Ma J., Li M., Cheng Y.T., Zhang Q., Wu D., Zhang Z., Zhang Y. (2016). Two redundant receptor-like cytoplasmic kinases function downstream of pattern recognition receptors to regulate activation of sa biosynthesis. Plant Physiol..

[72] Desaki Y., Miyata K., Suzuki M., Shibuya N., Kaku H. (2018). Plant immunity and symbiosis signaling mediated by lysm receptors. Innate Immun..

[73] Cao Y., Liang Y., Tanaka K., Nguyen C.T., Jedrzejczak R.P., Joachimiak A., Stacey G. (2014). The kinase lyk5 is a major chitin receptor in arabidopsis and forms a chitin-induced complex with related kinase cerk1. Elife.

[74] Yamada K., Yamaguchi K., Shirakawa T., Nakagami H., Mine A., Ishikawa K., Fujiwara M., Narusaka M., Narusaka Y., Ichimura K. (2016). The arabidopsis cerk1-associated kinase pbl27 connects chitin perception to mapk activation. EMBO J..

[75] Kaku H., Nishizawa Y., Ishii-Minami N., Akimoto-Tomiyama C., Dohmae N., Takio K., Minami E., Shibuya N. (2006). Plant cells recognize chitin fragments for defense signaling through a plasma membrane receptor. Proc. Natl. Acad. Sci. USA.

[76] Fan J., Bai P., Ning Y., Wang J., Shi X., Xiong Y., Zhang K., He F., Zhang C., Wang R. (2018). The monocot-specific receptor-like kinase sds2 controls cell death and immunity in rice. Cell Host Microbe.

[77] Lu D., Lin W., Gao X., Wu S., Cheng C., Avila J., Heese A., Devarenne T.P., He P., Shan L. (2011). Direct ubiquitination of pattern recognition receptor fls2 attenuates plant innate immunity. Science.

[78] Liao D., Cao Y., Sun X., Espinoza C., Nguyen C.T., Liang Y., Stacey G. (2017). Arabidopsis e3 ubiquitin ligase plant u-box13 (pub13) regulates chitin receptor lysin motif receptor kinase5 (lyk5) protein abundance. New Phytol..

[79] Wang Y.S., Pi L.Y., Chen X., Chakrabarty P.K., Jiang J., De Leon A.L., Liu G.Z., Li L., Benny U., Oard J. (2006). Rice xa21 binding protein 3 is a ubiquitin ligase required for full xa21-mediated disease resistance. Plant Cell.

[80] Nagashima Y., von Schaewen A., Koiwa H. (2018). Function of n-glycosylation in plants. Plant Sci..

[81] Haweker H., Rips S., Koiwa H., Salomon S., Saijo Y., Chinchilla D., Robatzek S., von Schaewen A. (2010). Pattern recognition receptors require n-glycosylation to mediate plant immunity. J. Biol. Chem..

[82] Li J., Zhao-Hui C., Batoux M., Nekrasov V., Roux M., Chinchilla D., Zipfel C., Jones J.D. (2009). Specific er quality control components required for biogenesis of the plant innate immune receptor efr. Proc. Natl. Acad. Sci. USA.

[83] Nekrasov V., Li J., Batoux M., Roux M., Chu Z.H., Lacombe S., Rougon A., Bittel P., Kiss-Papp M., Chinchilla D. (2009). Control of the pattern-recognition receptor efr by an er protein complex in plant immunity. EMBO J..

[84] Saijo Y., Tintor N., Lu X., Rauf P., Pajerowska-Mukhtar K., Haweker H., Dong X., Robatzek S., Schulze-Lefert P. (2009). Receptor quality control in the endoplasmic reticulum for plant innate immunity. EMBO J..

[85] Meng X., Zhang S. (2013). Mapk cascades in plant disease resistance signaling. Ann. Rev. Phytopathol..

[86] Zhang M., Su J., Zhang Y., Xu J., Zhang S. (2018). Conveying endogenous and exogenous signals: Mapk cascades in plant growth and defense. Curr. Opin. Plant Biol..

[87] Mizoguchi T., Ichimura K., Irie K., Morris P., Giraudat J., Matsumoto K., Shinozaki K. (1998). Identification of a possible map kinase cascade in arabidopsis thaliana based on pairwise yeast two-hybrid analysis and functional complementation tests of yeast mutants. FEBS Lett..

[88] Huang Y., Li H., Gupta R., Morris P.C., Luan S., Kieber J.J. (2000). Atmpk4, an arabidopsis homolog of mitogen-activated protein kinase, is activated in vitro by atmek1 through threonine phosphorylation. Plant physiol..

[89] Petersen M., Brodersen P., Naested H., Andreasson E., Lindhart U., Johansen B., Nielsen H.B., Lacy M., Austin M.J., Parker J.E. (2000). Arabidopsis map kinase 4 negatively regulates systemic acquired resistance. Cell.

[90] Suarez-Rodriguez M.C., Adams-Phillips L., Liu Y., Wang H., Su S.H., Jester P.J., Zhang S., Bent A.F., Krysan P.J. (2007). Mekk1 is required for flg22-induced mpk4 activation in arabidopsis plants. Plant Physiol..

[91] Li B., Jiang S., Yu X., Cheng C., Chen S., Cheng Y., Yuan J.S., Jiang D., He P., Shan L. (2015). Phosphorylation of trihelix transcriptional repressor asr3 by map kinase4 negatively regulates arabidopsis immunity. Plant Cell.

[92] Sun T., Nitta Y., Zhang Q., Wu D., Tian H., Lee J.S., Zhang Y. (2018). Antagonistic interactions between two map kinase cascades in plant development and immune signaling. EMBO Rep..

[93] Asai T., Tena G., Plotnikova J., Willmann M.R., Chiu W.L., Gomez-Gomez L., Boller T., Ausubel F.M., Sheen J. (2002). Map kinase signalling cascade in arabidopsis innate immunity. Nature.

[94] Zhang Z., Liu Y., Huang H., Gao M., Wu D., Kong Q., Zhang Y. (2017). The nlr protein summ2 senses the disruption of an immune signaling map kinase cascade via crck3. EMBO Rep..

[95] Lumbreras V., Vilela B., Irar S., Sole M., Capellades M., Valls M., Coca M., Pages M. (2010). Mapk phosphatase mkp2 mediates disease responses in arabidopsis and functionally interacts with mpk3 and mpk6. Plant J. Cell Mol. Biol..

[96] Jiang L., Anderson J.C., Gonzalez Besteiro M.A., Peck S.C. (2017). Phosphorylation of arabidopsis map kinase phosphatase 1 (mkp1) is required for pamp responses and resistance against bacteria. Plant Physiol..

[97] Bartels S., Gonzalez Besteiro M.A., Lang D., Ulm R. (2010). Emerging functions for plant map kinase phosphatases. Trends Plant Sci..

[98] Fuchs S., Grill E., Meskiene I., Schweighofer A. (2013). Type 2c protein phosphatases in plants. FEBS J..

[99] Cui F., Sun W., Kong X. (2018). Rlcks bridge plant immune receptors and mapk cascades. Trends Plant Sci..

[100] Shinya T., Yamaguchi K., Desaki Y., Yamada K., Narisawa T., Kobayashi Y., Maeda K., Suzuki M., Tanimoto T., Takeda J. (2014). Selective regulation of the chitin-induced defense response by the arabidopsis receptor-like cytoplasmic kinase pbl27. Plant J. Cell Mol. Biol..

[101] Liu T., Liu Z., Song C., Hu Y., Han Z., She J., Fan F., Wang J., Jin C., Chang J. (2012). Chitin-induced dimerization activates a plant immune receptor. Science.

[102] Bi G., Zhou Z., Wang W., Li L., Rao S., Wu Y., Zhang X., Menke F.L.H., Chen S., Zhou J.M. (2018). Receptor-like cytoplasmic kinases directly link diverse pattern recognition receptors to the activation of mitogen-activated protein kinase cascades in arabidopsis. Plant Cell.

[103] Yamada K., Yamaguchi K., Yoshimura S., Terauchi A., Kawasaki T. (2017). Conservation of chitin-induced mapk signaling pathways in rice and arabidopsis. Plant Cell Physiol..

[104] Wang C., Wang G., Zhang C., Zhu P., Dai H., Yu N., He Z., Xu L., Wang E. (2017). Oscerk1-mediated chitin perception and immune signaling requires receptor-like cytoplasmic kinase 185 to activate an mapk cascade in rice. Mol. Plant.

[105] Tsuda K., Somssich I.E. (2015). Transcriptional networks in plant immunity. New Phytol..

[106] Allen M.D., Yamasaki K., Ohme-Takagi M., Tateno M., Suzuki M. (1998). A novel mode of DNA recognition by a beta-sheet revealed by the solution structure of the gcc-box binding domain in complex with DNA. EMBO J..

[107] Li X., Duan X., Jiang H., Sun Y., Tang Y., Yuan Z., Guo J., Liang W., Chen L., Yin J. (2006). Genome-wide analysis of basic/helix-loop-helix transcription factor family in rice and arabidopsis. Plant Physiol..

[108] Dubos C., Stracke R., Grotewold E., Weisshaar B., Martin C., Lepiniec L. (2010). Myb transcription factors in arabidopsis. Trends Plant Sci..

[109] Ooka H., Satoh K., Doi K., Nagata T., Otomo Y., Murakami K., Matsubara K., Osato N., Kawai J., Carninci P. (2003). Comprehensive analysis of nac family genes in oryza sativa and arabidopsis thaliana. DNA Res. Int. J. Rapid Publ. Rep. Genes Genomes.

[110] Rushton P.J., Somssich I.E., Ringler P., Shen Q.J. (2010). Wrky transcription factors. Trends Plant Sci..

[111] Jakoby M., Weisshaar B., Droge-Laser W., Vicente-Carbajosa J., Tiedemann J., Kroj T., Parcy F., bZIP Research Group (2002). Bzip transcription factors in arabidopsis. Trends Plant Sci..

[112] Vailleau F., Daniel X., Tronchet M., Montillet J.L., Triantaphylides C., Roby D. (2002). A r2r3-myb gene, atmyb30, acts as a positive regulator of the hypersensitive cell death program in plants in response to pathogen attack. Proc. Natl. Acad. Sci. USA.

[113] Marino D., Froidure S., Canonne J., Khaled S.B., Khafif M., Pouzet C., Jauneau A., Roby D., Rivas S. (2019). Addendum: Arabidopsis ubiquitin ligase miel1 mediates degradation of the transcription factor myb30 weakening plant defence. Nat. Commun..

[114] Marino D., Froidure S., Canonne J., Ben Khaled S., Khafif M., Pouzet C., Jauneau A., Roby D., Rivas S. (2013). Arabidopsis ubiquitin ligase miel1 mediates degradation of the transcription factor myb30 weakening plant defence. Nat. Commun..

[115] Cheng M.C., Hsieh E.J., Chen J.H., Chen H.Y., Lin T.P. (2016). Correction. Arabidopsis rglg2, functioning as a ring e3 ligase, interacts with aterf53 and negatively regulates the plant drought stress response. Plant Physiol..

[116] Miao Y., Laun T., Zimmermann P., Zentgraf U. (2004). Targets of the wrky53 transcription factor and its role during leaf senescence in arabidopsis. Plant Mol. Biol..

[117] Miao Y., Zentgraf U. (2010). A hect e3 ubiquitin ligase negatively regulates arabidopsis leaf senescence through degradation of the transcription factor wrky53. Plant J. Cell Mol. Biol..

[118] Matsushita A., Inoue H., Goto S., Nakayama A., Sugano S., Hayashi N., Takatsuji H. (2013). Nuclear ubiquitin proteasome degradation affects wrky45 function in the rice defense program. Plant J. Cell Mol. Biol..

[119] Choi C., Hwang S.H., Fang I.R., Kwon S.I., Park S.R., Ahn I., Kim J.B., Hwang D.J. (2015). Molecular characterization of oryza sativa wrky6, which binds to w-box-like element 1 of the oryza sativa pathogenesis-related (pr) 10a promoter and confers reduced susceptibility to pathogens. New Phytol..

[120] Lee H., Cha J., Choi C., Choi N., Ji H.S., Park S.R., Lee S., Hwang D.J. (2018). Rice wrky11 plays a role in pathogen defense and drought tolerance. Rice.

[121] Mao G., Meng X., Liu Y., Zheng Z., Chen Z., Zhang S. (2011). Phosphorylation of a wrky transcription factor by two pathogen-responsive mapks drives phytoalexin biosynthesis in arabidopsis. Plant Cell.

[122] Andreasson E., Jenkins T., Brodersen P., Thorgrimsen S., Petersen N.H., Zhu S., Qiu J.L., Micheelsen P., Rocher A., Petersen M. (2005). The map kinase substrate mks1 is a regulator of plant defense responses. EMBO J..

[123] Qiu J.L., Fiil B.K., Petersen K., Nielsen H.B., Botanga C.J., Thorgrimsen S., Palma K., Suarez-Rodriguez M.C., Sandbech-Clausen S., Lichota J. (2008). Arabidopsis map kinase 4 regulates gene expression through transcription factor release in the nucleus. EMBO J..

[124] Menke F.L., Kang H.G., Chen Z., Park J.M., Kumar D., Klessig D.F. (2005). Tobacco transcription factor wrky1 is phosphorylated by the map kinase sipk and mediates hr-like cell death in tobacco. Mol. Plant Microbe Interact..

[125] Jiao Y., Wang Y., Xue D., Wang J., Yan M., Liu G., Dong G., Zeng D., Lu Z., Zhu X. (2010). Regulation of osspl14 by osmir156 defines ideal plant architecture in rice. Nat. Genet..

[126] Withers J., Dong X. (2016). Posttranslational modifications of npr1: A single protein playing multiple roles in plant immunity and physiology. PLoS Pathog..

[127] Zhang X., Chen S., Mou Z. (2010). Nuclear localization of npr1 is required for regulation of salicylate tolerance, isochorismate synthase 1 expression and salicylate accumulation in arabidopsis. J. Plant Physiol..

[128] Kinkema M., Fan W., Dong X. (2000). Nuclear localization of npr1 is required for activation of pr gene expression. Plant Cell.

[129] Mou Z., Fan W., Dong X. (2003). Inducers of plant systemic acquired resistance regulate npr1 function through redox changes. Cell.

[130] Fu Z.Q., Yan S., Saleh A., Wang W., Ruble J., Oka N., Mohan R., Spoel S.H., Tada Y., Zheng N. (2012). Npr3 and npr4 are receptors for the immune signal salicylic acid in plants. Nature.

[131] Spoel S.H., Mou Z., Tada Y., Spivey N.W., Genschik P., Dong X. (2009). Proteasome-mediated turnover of the transcription coactivator npr1 plays dual roles in regulating plant immunity. Cell.

[132] Saleh A., Withers J., Mohan R., Marques J., Gu Y., Yan S., Zavaliev R., Nomoto M., Tada Y., Dong X. (2015). Posttranslational modifications of the master transcriptional regulator npr1 enable dynamic but tight control of plant immune responses. Cell Host Microbe.

[133] Wang D., Amornsiripanitch N., Dong X. (2006). A genomic approach to identify regulatory nodes in the transcriptional network of systemic acquired resistance in plants. PLoS Pathog..

[134] Despres C., DeLong C., Glaze S., Liu E., Fobert P.R. (2000). The arabidopsis npr1/nim1 protein enhances the DNA binding activity of a subgroup of the tga family of bzip transcription factors. Plant Cell.

[135] Ning Y., Liu W., Wang G.L. (2017). Balancing immunity and yield in crop plants. Trends Plant Sci..

[136] Wang J., Zhou L., Shi H., Chern M., Yu H., Yi H., He M., Yin J., Zhu X., Li Y. (2018). A single transcription factor promotes both yield and immunity in rice. Science.

